# Modeling trends of health and health related indicators in Ethiopia (1995-2008): a time-series study

**DOI:** 10.1186/1478-4505-7-29

**Published:** 2009-12-13

**Authors:** Mulu W Abraha, Tilahun H Nigatu

**Affiliations:** 1 Health Promotion and Disease prevention Directorate, Federal Ministry of Health, Addis Ababa, Ethiopia; 2 HIV/AIDS, Population and Nutrition office, United States Agency for International Development/USAID, Addis Ababa, Ethiopia

## Abstract

**Background:**

The Federal Ministry of Health of Ethiopia has been publishing Health and Health related indicators of the country annually since 1987 E.C. These indicators have been of high importance in indicating the status of health in the country in those years. However, the trends/patterns of these indicators and the factors related to the trends have not yet been investigated in a systematic manner. In addition, there were minimal efforts to develop a model for predicting future values of Health and Health related indicators based on the current trend.

**Objectives:**

The overall aim of this study was to analyze trends of and develop model for prediction of Health and Health related indicators. More specifically, it described the trends of Health and Health related indicators, identified determinants of mortality and morbidity indicators and developed model for predicting future values of MDG indicators.

**Methods:**

This study was conducted on Health and Health related indicators of Ethiopia from the year 1987 E.C to 2000 E.C. Key indicators of Mortality and Morbidity, Health service coverage, Health systems resources, Demographic and socio-economic, and Risk factor indicators were extracted and analyzed. The trends in these indicators were established using trend analysis techniques. The determinants of the established trends were identified using ARIMA models in STATA. The trend-line equations were then used to predict future values of the indicators.

**Results:**

Among the mortality indicators considered in this study, it was only Maternal Mortality Ratio that showed statistically significant decrement within the study period. The trends of Total Fertility Rate, physician per 100,000 population, skilled birth attendance and postnatal care coverage were found to have significant association with Maternal Mortality Ratio trend. There was a reversal of malaria parasite prevalence in 1999 E.C from *Plasmodium Falciparum *to *Plasmodium Vivax*. Based on the prediction from the current trend, the Millennium Development Goal target for under-five mortality rate and proportion of people having access to basic sanitation can be achieved.

**Conclusion:**

The current trend indicates the need to accelerate the progress of the indicators to achieve MDGs at or before 2015, particularly for Maternal Health and access to safe water supply.

## Introduction

There is no single "standard" measurement of health status for individuals or population groups. Individual health status may be measured by an observer (e.g., a physician), who performs an examination and rates the individual along any of several dimensions, including presence or absence of life-threatening illness, risk factors for premature death, severity of disease, and overall health. Individual health status may also be assessed by asking the person to report his/her health perceptions in the domains of interest, such as physical functioning, emotional well-being, pain or discomfort, and overall perception of health [1].

The Health of an entire population is determined by aggregating data collected on individuals. The health of an individual is easier to define than the health of a population. Once the definition of optimum health for the individual is agreed upon, health status can be placed along a continuum from perfect health to death. No comparable scale exists for whole populations. What is the population-level equivalent of death? (Keep in mind that it is unusual for entire populations to die.) In the absence of comprehensive or absolute measures of the health of a population, the average lifespan, the prevalence of preventable diseases or deaths, and availability of health services serve as indicators of health status [2].

Hence, judgments regarding the level of health of a particular population are usually made by comparing one population to another, or by studying the trends in a health indicator within a population over time. There are two main goals of time-series analysis (a) identifying the nature of the phenomenon represented by the sequence of observations, and (b) forecasting (predicting future values of the time series variable). Both of these goals require that the pattern of observed time series data is identified and more or less formally described. Once the pattern is established, we can interpret and integrate it with other data [3].

One of the hallmarks of epidemiologic analysis is the understanding that health outcomes in a population can only be fully understood if their frequency and distribution is examined in terms of person, place, and time. Trend analysis is one leg of this analytic triangle, and is used for public health surveillance and monitoring, for forecasting, for program evaluation, for policy analysis, and for etiologic analysis, investigation of potentially causal relationships between risk factors and outcomes [4].

The Federal Ministry of Health of Ethiopia has been publishing Health and Health related indicators as a stand-alone document since 1987 E.C. Through this document, which is published on annual basis, the annual status of several Health and Health related indicators is reported. Mortality and morbidity indicators, Health service coverage indicators, Health system resource indicators and socio-economic indicators are the major ones [5].

Though the Ministry has been producing such useful indicators the trend of those important indicators hasn't been systematically investigated. This doesn't however mean that there are no efforts in analyzing the trends. There are efforts in presenting trends through graphs and tables within the indicator documents. But as to the existing knowledge, there are no studies that tried to fit the trends in modeling equations and predict future values based on the current trends for the majority of the indicators. Besides, the determinants of changes in key health status indicators like Maternal Mortality Ratio and Infant Mortality Rate hasn't been established through modeling exercises and regression models in a time-series dataset of the indicators [6].

In light of this, the envisaged study was designed to describe the overall pattern of Health and Health related indicators overtime by comparing one time-period to another time-period in the series. The study had also developed trend-line equations/models that can be used to predict future values based on the current trend. Moreover, these equations/models were used to predict MDG indicator values for 2015 which were then compared with their target values. Among the purposes was also identification of determinants of changes/improvements in mortality and morbidity indicators during the study period. This study therefore has identified determinants of mortality and morbidity indicators in Ethiopia during the period of 1987 E.C to 2000 E.C (1995 to 2008) [7].

## Methods

### Study area and period

This study was conducted on Health and Health related indicators of Ethiopia. The current population is approximately 73.9 million, of which 83.86 percent live in rural areas. Ethiopia is a Federal Democratic Republic composed of 9 National Regional states two Administrative states. The national regional states as well as the two cities administrative councils are further divided into six hundred eleven woredas and around 15,000 kebeles (5,000 Urban & 10,000 Rural). This study was conducted from January 2001 E.C to May 2001 E.C.

### Study design and indicators

This study followed the time-series type of study design based on the review of annual reports of Health and Health related indicators in Ethiopia. The source indicators for this study were all Health and Health related indicators in Ethiopia. The units of analysis of this study were Health and Health related indicators in Ethiopia for 14 years period (1987-2000 E.C). Accordingly, the study indicators were selected Health and Health related indicators in Ethiopia.

### Sample and sampling

The number of time-periods being examined for each health and health related indicator was 14 (1987-2000 E.C), that is since the time at which Health and Health related indicators were obtained. The number of Health and Health related indicators considered in this study was selected in the following way. First, the Health and Health related indicators were categorized in to five major categories based on the WHO 2008 health statistics.

The categories and the number of indicators in each category were Mortality and Morbidity indicators (14 indicators), Health service coverage indicators (10 indicators), Health systems resources indicators (10 indicators), Demographic and socio-economic indicators (10 indicators), and Risk factors indicators (6 indicators). These all make the total number of indicators included in the study to be 50.

From each category of Health & Health related indicator, study indicators were selected purposively. The criteria that were used to select the indicators were: Public Health importance/widely used, data availability for the study period, measurement at national, relevance to MDGs and presence/absence of significant measurement change during the study period.

A total of 90 indicator documents were reviewed of which 45 national and the rest 45 were international documents. Indicator values were cross-evaluated across and within these sources.

For the purpose of this study, three instruments were employed: Documents collection checklists, indicator collection format and indicator definition. In order to collect the documents from which study indicators and/or their determinant obtained a document collection checklist was used to facilitate the collection of indicator documents. An excel sheet to which the values of study indicators were filled was used to collect the relevant indicators of the study across the study duration. This definition was based on the definition of Ministry of Health and other sectors. The indicator definition was prepared (adopted) to assist the interpretation of the findings.

Six Public Health Professionals were oriented about the study and its data requirements based on the objective of the study. They collected the available documents (in soft and/or hard copies) that bear indicator values and they referred for the rest of the documents to departments of the Ministries (Authorities) and libraries. The principal investigator reviewed the collected documents and extracted the indicators.

### Data analysis and interpretation

All the indicator values were entered in to a spreadsheet in Ms-excel. The entered data were checked once again for accuracy of data entry. Errors during the entry were cleaned and analysis started. The analysis of the data followed the following steps:

#### Describing Trends

The first step in the analysis looked in to trend in each Health and Health related indicator. The patterns of each Health and Health related indicator were described numerically and graphically. The changes of the indicators were also described using numerical summaries and line graphs. Changes of mortality and morbidity indicators were additionally described by confidence intervals using STATA 8.0 and only those with statistically significant changes, non-overlapping confidence intervals were considered for Auto Regressive Iintegarted Moving Average (ARIMA) model.

#### Fitting trend-line equations

The trend was plotted using a line graph by connecting the points on the X-Y axis, where X is the time in year and Y is value of the selected indicator for each year. Then an effort was made to identify a trend-line equation that best fits the line using coefficient of determination (R^2^). Data transformation and smoothing was made to change the rate and flatten the series of rates without changing the shape of the trend (e.g. MMR≅ log MMR). The rate of annual change of the indicator values (R) was estimated by equating the indicator (I) and time (T): **I = B0 + R (T), **when linearity was assumed. If linearity was not assumed, other trend-line options (trend/regression types) including polynomial, logarithmic, exponential and power were considered

#### Identifying determinants

Here morbidity and mortality indicators were considered as outcome variables and any indicator that affected the outcome variables was taken as independent variables. For the given period of 14 years, the morbidity (burden of disease) and mortality indicators which showed statistically significant change were assessed for their association with possible determinants (based on literature evidence) which had statistically significant change across time. First correlation of variables was observed in a correlation matrix. Then all potential determinants of a dependent variable (indicator) were fitted in to ARIMA (Auto Regression Integrated Moving Average) model in STATA 8.0. For all cases, P-values < 0.05 was considered to be statistically significant.

#### Predicting future values

The best fit equations of the trend-line of an indicator were used to predict the future values of the indicator for 2015. These predicted values were then compared with the targets for 2015 by the MDG. Then the findings were discussed in the perspective of achieving the MDGs.

#### Ethical consideration

Ethical clearance was obtained from the Ethical review committee of Faculty of Health, Jimma University. Official permission was requested from the different organizations for documents. For data obtained from publications and web pages, all the data sources were acknowledged.

## Results

### 1. Description of trends

#### Mortality indicators

In this study five major mortality indicators were used: Infant Mortality Rate (IMR), Maternal Mortality Ratio (MMR), Under-five Mortality Rate (U5MR), Crude Death Rate (CDR), and Life Expectancy at Birth (LEB). These indicators were measured in 1994 census, DHS 2000 and DHS 2005. Hence these indicators have only three measurement values. The mortality indicators and their 95% confidence intervals are shown in Table 1.

**Table 1:** Mortality indicators of Ethiopia and their 95% confidence intervals, Ethiopia

Indicators	1994 census	DHS 2000	DHS 2005
IMR	110 [90.6, 129.3]	97 [78.6, 115.3]	77 [60.5, 93.5]

MMR	1400 [1327, 1473]	871 [813, 928]	673 [622, 723]

U5MR	161 [138.2, 183.8]	140.1 [118.6, 161.6]	123 [102.6, 143.4]

CDR	14.96 [7.4, 22.5]	12.6 [5.7, 19.5]	10.7 [4.3, 17.1]

LEB Female	53	55.4	55.4

LEB Male	50.9	53.4	53.4

As shown in Table 1, the 95% confidence intervals of the mortality indicators overlap each other except for Maternal Mortality Ratio. For example the 1994 census Infant Mortality Rate value was 110 with the confidence interval of 90.6 and 129.3, and the DHS 2000 value of Infant Mortality Rate was 97 with confidence interval of 78.6 and 115.3 which showed an overlap of values. The same is true for under-five Mortality Ratio and Crude Death Rate. This shows that it is only the Maternal Mortality that shown a statistically significant decrement in the study period. The rest of the mortality indicators showed a decrement but haven't shown any statistically significant reduction.

The decrease in the mortality rates (maternal mortality rate, infant mortality rate, and under-five mortality rates) during the study period are shown in the following graph. The higher change (decrement) of Infant mortality rate was occurred between years 1987 to 1993 however for Maternal Mortality Ratio, Under-five Mortality rate and Crude Death Rate greater change was shown between 1987 and 1993 E.C. The highest decrease in mortality was the under-five mortality rate followed by the infant mortality rate. The least decrement is observed in Crude Death Rate followed by Maternal Mortality Ratio.

**Figure 1:**
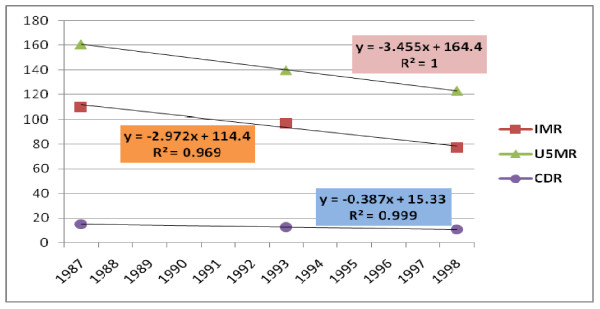
**Trends in IMR, U5MR, and CDR of Ethiopia**.

**Figure 2:**
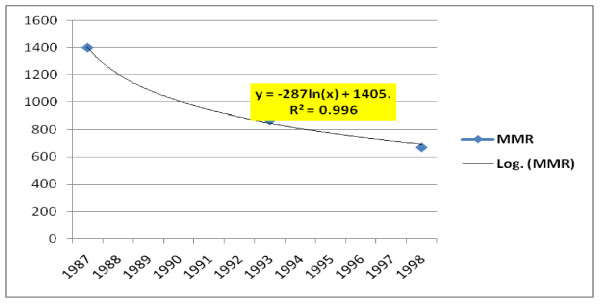
**Trends in MMR of Ethiopia**.

In analysis of trends in the major mortality indicators, infant mortality rate, under-five mortality rate, and crude death rate were found to have linear decrement trend. The yearly decrement rates for these indicators were 2.97 per 100,000 live births, 3.45 per 100,000 live births and 0.38 per 1000 population respectively (Figure 1). However, the Maternal Mortality Ratio followed the logarithmic trend with a yearly decrement rate of 672 per 100,000 live births (Figure 2).

**Figure 3:**
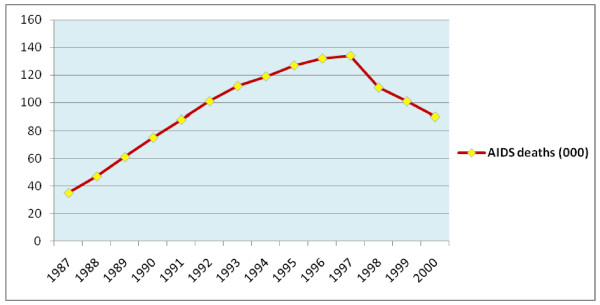
**Trends in estimated AIDS deaths from 1987-2000 E.C, Ethiopia**.

With regard to AIDS related mortality, there were an estimated total of 1.3 million AIDS deaths in the study period (1987-2000). This will make median AIDS related deaths per year to be 101,000. The maximum estimated number of AIDS deaths per year was 134 thousand which was observed in 1997. The trend of AIDS related deaths had an increasing trend but it showed a decrement after 1997 (Figure 3).

As to Tuberculosis related mortality, there were 17,022 reported Tuberculosis deaths in the period 1992-2000. The median number of TB deaths per year was found to be 1993 with a range of 898 to 2548. The smallest number of TB deaths was observed in 1992 while the largest was in 1997. The pattern of TB deaths had an increasing trend till 1997 but a decreasing pattern after 1997.

**Figure 4:**
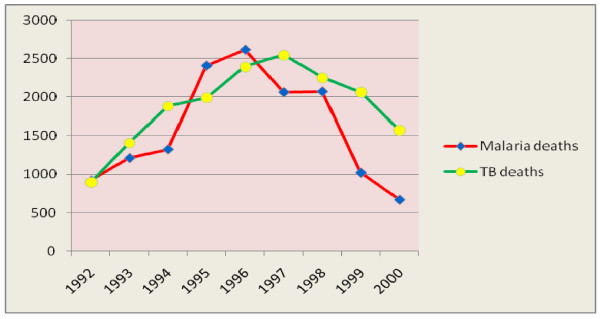
**Trend in Malaria and TB deaths from 1992-2000 E.C, Ethiopia**.

In the period 1992-2000 there were 14303 reported *Malaria *deaths. The median number of malaria deaths per year was 1325. The minimum and maximum number of malaria deaths was 669 and 2616 which were reported in 2000 and 1996 E.C respectively. Number of malaria deaths per year had an increasing trend from 1992 to 1996 and a decreasing trend from 1996 to 2000 E.C (Figure 4).

#### Morbidity indicators

In the years 1991-2000 E.C, the median number of total outpatient visits was 19.5 million per year. The minimum and the maximum number of total outpatient visits were 260 and 360 per 1000 population reported in 2000 and 1996 E.C respectively.

The median number of inpatients in years 1992-2000 E.C was about 0.4 million per year (530 per 100,000 population per year). The minimum and maximum number of admission per year was 158 and 1510 per 100,000 population which were reported in 1992 and 1997 respectively. The median number of inpatients per 1000 outpatient visits was 18 with a range of 4.5 in 1996 to 50.5 in 1997.

**Figure 5:**
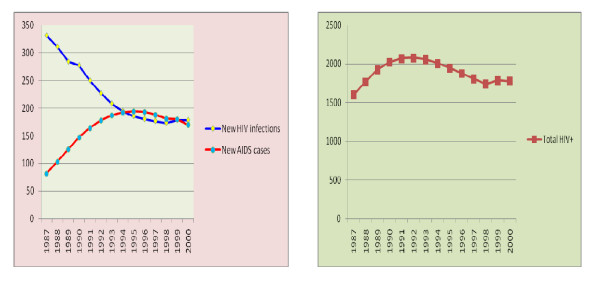
**A) Trends in new HIV infections and new AIDS cases, B) Trends in total HIV positive people**.

The incidence rate for HIV from 1987 to 2000 E.C had a median of 201.5 per 100,000 population per year. The number of new HIV infections per 100,000 population per year ranges from 173 in1998 to 331 in 1987 E.C. The Prevalence rate of HIV in the study period had a median value of 1900 per 100,000 population with a range of 1600 in 1987 to 2079 in 1992. The incidence of AIDS cases in the study period had a median of 179 per 100,000 population per year with a range of 82 in 1987 to 194 in 1995 E.C (Figure 5)

**Figure 6:**
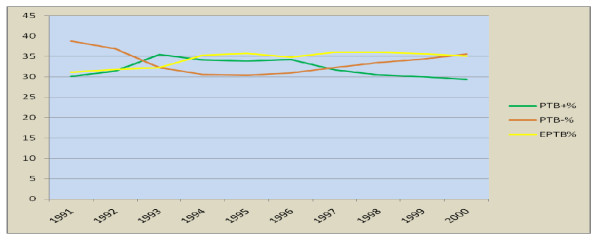
**Trends in PTB+, PTB- and EPTB from 1991-2000 E.C, Ethiopia**.

The median number of new TB cases per 100,000 population in the period 1992-2000 was 158 while that of all TB cases was 162. The new cases range from 131 to 183 per 100000 population while the number of total TB cases ranged from 133 to 187 per 100000 population per year. Minimum rates were observed in 1987 while the maximum in 2000. There was an increasing trend till 1996 and a decrement in 1997 and 1998 but again an increase to above the previous higher level in 1999 and 2000. The trend in smear positive pulmonary TB, smear negative pulmonary TB, and extra-pulmonary TB, have increasing trend for smear negative PTB and Extra-pulmonary TB. There is a decreasing in smear positive pulmonary TB(Figure 6).

**Figure 7:**
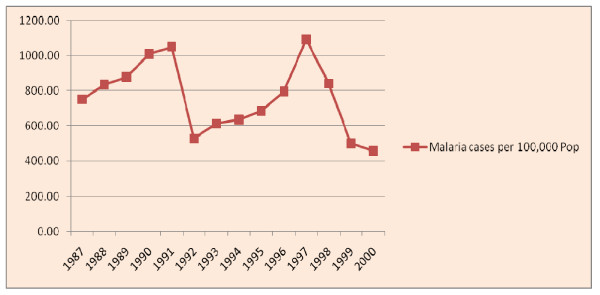
**The trend of malaria Positive cases per 100,000 population per year**.

With regard to malaria morbidity, there were about 7 million malaria positive cases in the study period (1987-2000 E.C). This will make an average of half a million malaria positive cases per year. There were two peaks in the number of malaria cases. The smaller peak was in 1991 and the higher peak was in 1997 E.C. Lower number cases were reported in 1992 and the lowest was in 2000. There was a dramatic decrease in malaria as of 1997 E.C. The median number of malaria positive cases per year was 773 per 100,000 population with a minimum of 457 in 2000 and a maximum of 1092 in 1997 E.C. The median number of P. Falciparum and P. Vivax cases per 100,000 population per year in the same period was 493 and 249 respectively (Figure 7).

**Figure 8:**
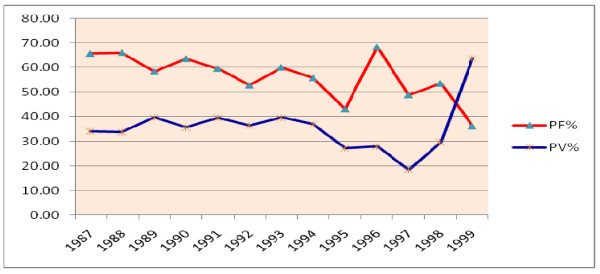
**The percentage contribution of P. Falciparum and P. Vivax to all malaria cases**.

There was a reversal of malaria parasite prevalence in 1999 E.C from P. Falciparum to P. Vivax. P. Falciparum remained to be the major prevalent species till 1998. However, in 1999 this leading role was overtaken by P. Vivax. In 1987 E.C, the percentage of P. Falciparum and P. Vivax were 65.6 and 34.3 respectively. But in 1999 E.C, the percentage for P. Falciparum and P. Vivax were 36.3 and 63.7 respectively. This shows the reversal of the parasite type in Ethiopia (Figure 8).

**Figure 9:**
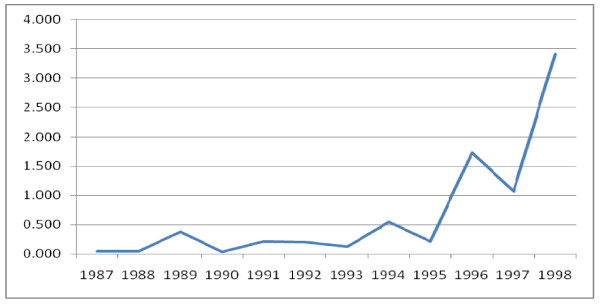
**Trends of mixed infection of malaria from 1987-1998 E.C, Ethiopia**.

Regarding the percentage of mixed infection (P. Falciparum and P. Vivax) there was almost non/few mixed infections at 1987 (0.046%) but gradually the mixed infection had an increasing trend which reached peak in 1998 (3.4%) (Figure 9).

#### Health service coverage indicators

To describe the Health service coverage, Antenatal care (ANC), Skilled birth attendance (SBA), Postnatal care (PNC), Contraceptive prevalence rate (CPR), Expanded Program for Immunization coverage (EPI), and Potential Health Service coverage from the government facilities (PHS) were used. There is a general exponentially increasing trend in all these Health service coverage indicators during the study period. The total increase and the average increase per year are shown in Table 2.

**Table 2:** The total increase and average increase per year in Health service coverage indicators

	ANC	SBA	PNC	CPR	EPI	PHS
Total increase (%)	41.20	16.10	18.40	51.60	71.40	44.60

Average increase/year	3.17	1.24	1.42	3.97	5.49	3.43

**Figure 10:**
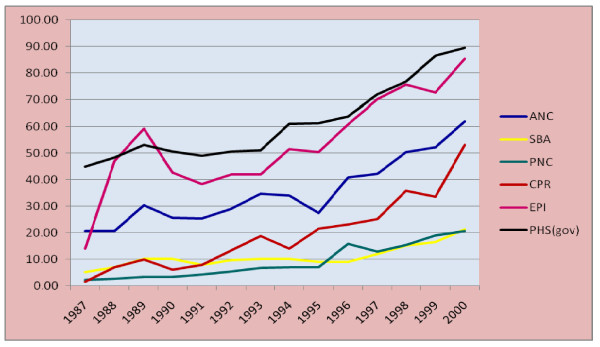
**Trends in Health service coverage indicators (1987-2000 E.C), Ethiopia**.

As shown in the above table, the highest increase in service coverage was on EPI coverage followed by CPR and Potential Health Service coverage. The lowest increment among the six Health service coverage indicators was on skilled birth attendance and postnatal care (Figure 10).

#### Health system resources indicators

**Figure 11:**
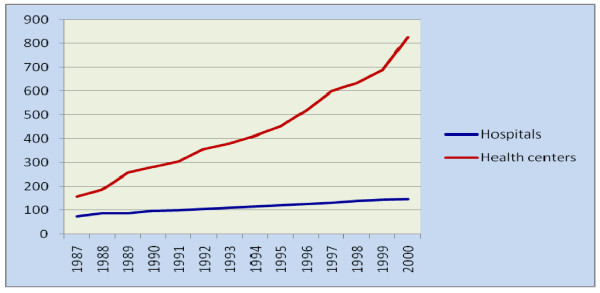
**The number of Hospitals and Health centers across the years (1987-2000 E.C), Ethiopia**.

**Figure 12:**
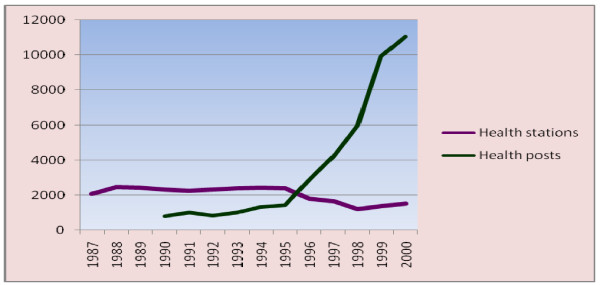
**The number of Health Posts and Health stations across the years (1987-2000 E.C), Ethiopia**.

During the study period the number of Hospitals increased from 73 to 146 (an average of 5.2 hospitals per year). During the same period the number of Health centers increased from 157 to 826 (an average of 51 Health centers per year).). The number of Health stations has decreased from 2450 to 1517 (73 Health station per year). The number of Health posts during the period of 1990-2000 E.C increased from 802 to 11031 (an average of 1136 Health posts per year) (Figure 11) (Figure 12).

The hospital to population ratio has increased from 1:752,577 in 1987 to 1:518,948 E.C in 2000 E.C. During the same period the Health centre to population ratio has increased from 1:349,924 to 1:91,726. In the period 1990-2000 E.C, the Health Post to population ratio has increased from 1:74,666 to 1: 6,868. The ratio of beds (Hospital beds and Health centre beds) to population has increased from 1:5,859 in 1988 to 1:5,198 in 2000 E.C. The mean number of beds per Health centre ranges from 1-6, while the mean number of beds per hospital ranges from 90-134.

**Figure 13:**
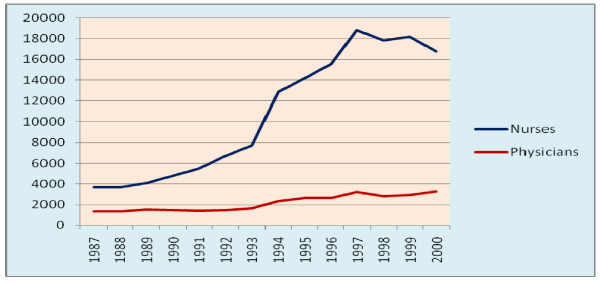
**Trends of number of nurses and physicians 1987-2000, Ethiopia**.

The number of both nurses and physicians has an increasing trend during the study period. The average rate of increment per year were 1000 and 150 for nurses and physicians respectively. The nurse to population ratio has increased from 1:14,784 to 1:4,519 and the physician to population ratio has increased from 1:40,277 to 1:22,918 (the WHO recommends nurse to population ratio of 1:5,000 and physician to population ratio of 1:10,000). The physician to nurse ratio has decreased from 1:2.72 in 1987 to 1:5.07 in 2000. The average hospital to physician ratio was 1:19 with a minimum of 1:25 in 1997 maximum of 1:14 in 1991 and 1992 (Figure 13).

The total health budget allocated has increased from about half a billion to 2 billion per year while the expenditure has increased from 0.4 to 1.6 billion making the average percent of the expenditure 80. The health budget per capita per year ranges from 10.24ETB to 27.57ETB with a mean (SD) of 16.39 (5.3) ETB. The average rate of increment in health budget per capita per year was 1.33ETB. Similarly, the health expenditure per capita per year ranges from 7.8 to 22.1ETB with a mean (SD) of 12.8(4.1) ETB. The averages rate of increment in health expenditure per capita per year was 1.1ETB.

#### Demographic and socioeconomic indicators

During the study period, the population of Ethiopia has increased from 53-73 million. The average increment rate per year was 1.54 million (2.91%). The total fertility rate in 1987 was 6.4 then it decreased to 5.5 in 1992 and to 5.4 in 1997. This is equivalent to a decrease of 1 child per woman during ten year period. The highest decline in TFR was in the period 1987-1992 E.C which is 0.9 as compared to 0.1 in the period 1993-1997 E.C. Crude birth rate was 44.2,39.9 and 35.7 per 1000 population in 1987, 1993 and 1997 respectively. It has a decrement of 8.47 per 1000 population within 10 years period.

**Figure 14:**
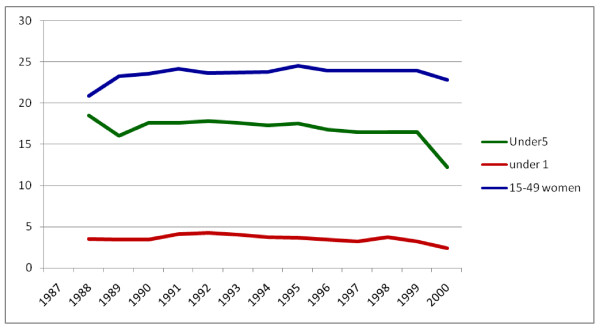
**Trends of proportion of Under-five, under one and 15-49 women in Ethiopia**.

The percentage of urban population during the study period has increased from 14.4% to 16.5%. This has an increment of 2.1%. Had the urban population proportion remained the same as it were in 1987, the current urban population would have been 10644265. But now it is 12196553 which have an increment of 1552289 populations. Under-five population has decreased from 18.5% in 1987 to 12.2% in 2000 which showed a total of 6.3% decrement with an average of 0.5% per year. Under-one population also showed a decrement of 1.14% within the study period. Regarding the proportion of women of reproductive age group it ranges from 22.8% to 24.5%. The trends of proportion of under-five, under-one and women of reproductive age group are shown in Figure 14.

Adult literacy rate (15 years and above) has increased from 27% in 1987 to 35.9% in 1992 and then to 64.9 in 1997. There is a total increment of 37.9% with 10 years period. The overall trend of increment of adult literacy rate followed an exponential function.

**Figure 15:**
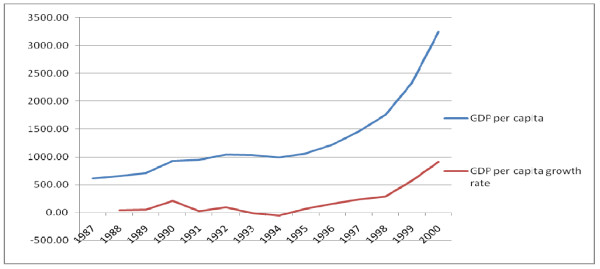
**Trends in GDP per capita per year and its growth rate 1987 to 2000, Ethiopia**.

Gross Domestic Product (GDP) at current market price has grown from 33.9 billion ETB in 1987 to 245.6 ETB billion in 2000. There was a total increment of 211.7 billion ETB with a mean (SD) increment rate of 16.3 (20.7) billion ETB per year. GDP per capita per year has increased from 616.8 ETB in 1987 to 3241.3 ETB in 2000. There was a total increment of 2624.5 ETB with a mean (SD) increment rate of 201.9 (269.8) ETB per year per capita (Figure 15).

Human development index (HDI) is a composite development indicator comprising of life expectancy at birth from health, GDP from economy and adult literacy rate from education. The Ethiopian HDI as reported by United Nations Development Program (UNDP) has increasing trend. Specific values are shown in Table 3.

**Table 3:** Trends in human development index (HDI) 1987 to 1997, Ethiopia

S#	Year	HDI values
1	1987	0.347

2	1992	0.379

3	1997	0.406

Source: UNDP 2008

#### Risk factor indicators

The average (SD) increment rates of access to safe water, piped water and basic sanitation were 20.2 (8.6), 5(1.4) and 15(7.1) respectively from 1987 to 1997. The pattern of these environmental health indicators is shown in Table 4.

**Table 4:** Trends of safe water supply and basic sanitation coverage, Ethiopia

S#	Risk factor indicators	1987 E.C	1992 E.C	1997 E.C
1	Access to safe water (%)	19.1	33.2	59.5

2	Access to piped water (%)	14	18	24

3	Access to basic sanitation (%)	8	18	38

Source: UNDP 2008

Prevalence of underweight and stunting of under-five had a decreasing trend with an average (SD) increment rate of 6.3(3.3) and 8.78(4.6) per five year respectively. In annual basis, the prevalence of underweight had a decrement rate of 1.26. However prevalence of wasting showed an increment of 2.5 from 1987 to 1992 and had no change for the subsequent five years (Table 5).

**Table 5:** Trends of prevalence of malnutrition among under-five children, Ethiopia

S#	Risk factor indicators	1987 E.C	1992 E.C	1997 E.C
1	Prevalence of underweight (%)	51	47	38.4

2	Prevalence of wasting (%)	7	10.5	10.5

3	Prevalence of Stunting (%)	64	52	46.5

Source: UNDP 2008

### 2. Determinants of Trends of Mortality and Morbidity Indicators

#### Maternal Mortality Ratio (MMR)

Twelve variables were found to have statistically significant correlation with Maternal Mortality Ratio. The variables, their correlation coefficients and significance level are shown in (Table 6).

**Table 6:** Correlation of Maternal Mortality Ratio with other covariates

S#	Covariates	Corr. coefficient	P-Value
1	GDP per capita	-0.7087	0.0045

2	Adult Literacy Rate	-0.8286	0.0003

3	Total Fertility Rate	0.9981	0.0000

4	Physicians per 100,000 Population	-0.7774	0.0011

5	Nurses per 100,000 Population	-0.8749	0.0000

6	Hospitals per 100,000 population	-0.9679	0.0000

7	Health Centres per 100,000 population	-0.9079	0.0000

8	Antenatal care coverage	-0.8041	0.0005

9	Skilled Birth Attendant	-0.7328	0.0029

10	Postnatal care coverage	-0.8144	0.0004

11	Contraceptive Prevalence rate	-0.8151	0.0004

12	Recurrent Health Expenditure per capita	-0.6835	0.0070

Source: UNDP 2008

The crude and adjusted beta-coefficients obtained using Autoregressive integrated moving average (ARIMA) model for the association of the above covariates with Maternal Mortality Ratio (MMR) are shown in (Table 7). As there were equal laggs on all the variables ARIMA is used to control for third variables that could explain the association.

**Table 7:** Crude and Adjusted β coefficients of determinants of Maternal Mortality Ratio with their CIs

S#	Covariates	Crude β	Adjusted β
1	GDP per capita	-0.22 [-0.32, -0.11]	-0.02 [-0.30, 0.26]

2	Adult Literacy Rate	-8.47 [-12.8, -4.4]	3.7 [-6.57, 14.0]

3	Total Fertility Rate	709.0 [689.4, 728.7]	723.6 [560.5, 886.6]

4	Physicians per 100,000 Pop	-211.2 [-370.2, -52.1]	40.8 [9.9, 71.8]

5	Nurses per 100,000 Population	-25.7 [-38.3, -13.1]	-13.8 [-35.5, 7.8]

6	Hospitals per 100,000 population	-12782 [-14601.2, -10963.3]	

7	Health Centres per 100,000 pop^n^	-875.6 [-1031.0, -720.1]	293 [-673.6, 1260]

8	Antenatal care coverage	-14.3 [-20.4, -8.2]	4.42 [-3.7, 12.5]

9	Skilled Birth Attendant	-39.2 [-57.6, -20.7]	-16.3 [-24.7, -7.86]

10	Postnatal care coverage	-28.2 [-47.1, -9.4 ]	-11.89 [-18.35, -5.43]

11	Contraceptive Prevalence rate	-12.8 [-16.5, -9.1]	-1.76 [-4.38, 0.86]

12	Recurrent Health Exp. per capita	-37.3 [-58.4, -16.2]	-3.46 [-8.95, 2.04]

13	Constant		-3736.46 [-4826.07, -2646.85]

* P-value < 0.05, statistically significant

In the final model, physician per 100,000 populations, skilled birth attendant and postnatal care coverage were found the only predictor time series variables that had statistically significant association with Maternal Mortality Ratio (Table 8).

**Table 8:** Condensed Model of Determinants of Maternal Mortality Ratio

S#	Covariates	Adjusted β
1	Total Fertility Rate	723.6 [560.5, 886.6]

2	Physicians per 100,000 Population	40.8 [9.9, 71.8]

3	Skilled Birth Attendant	-16.3 [-24.7, -7.86]

4	Postnatal care coverage	-11.89 [-18.35, -5.43]

5	Constant	-3736.46 [-4826.07, -2646.85]

* P-value < 0.05, statistically significant

As it mention in table 8, Total fertility rate (TFR) and physicians per 100,000 population are positively associated with Maternal Mortality Ratio, while skilled birth attendant and postnatal care coverage are negatively associated with the Maternal Mortality Ratio. As it is mentioned earlier, Infant Mortality Rate, Child Mortality rate, Crude death Rate have a list of determinants. However even though there a change within the study period but they have no statistically significant changes hence no modelling was anticipated to them.

### 3. Prediction of Health and Health Related Indicators

Based on the time-trend of the mortality and morbidity indicators in the past 14 years, the following equations with their respective R^2 ^were obtained. Using those equations the values of the indicators for 2015 (MDG target year) were predicted. As it is shown in the following table, during 2015 IMR, U5MR, MMR, CDR, MLE and FLE will have 52, 92, 531, 7, 54 and 56 per 1000 population respectively (Table 9).

**Table 9:** Predicted values of Major Mortality indicators for 2015, Ethiopia

Indicator	Equation	**R**^2^	By 2015 (t = 21)
IMR	IMR = -2.972t + 114.4	0.969	52

U5MR	U5MR = -3.455t + 164.4	0.999	92

MMR	MMR = -287ln(t) + 1405	0.996	531

CDR	CDR = -0.387t + 15.33	0.999	7

MLE	MLE = 1.08ln(t) + 50.97	0.98	54

FLE	FLE = 1.036ln(t) + 53.06	0.98	56

* P-value < 0.05, statistically significant

Similarly, the time-trend for Morbidity indicators with cumulative transformation was conducted. The equation for the cumulative rates with R^2 ^and projection for 2015 values were conducted (Table 10).

**Table 10:** Prediction of major morbidity indicators (Cumulative) by 2015, Ethiopia

Indicator	Equation	**R**^2^	By 2015 (t = 21)
TB cases/1000pop (Cumulative)	TBc = 1.68t+0.996	0.999	36.276

Malaria cases/1000 (Cumulative)	Mc = 7.586t+10.98	0.994	170.286

OPD per 1000 pop (cumulative)	OPD = 295t+165.8	0.995	6360.8

IPD per 1000 pop (Cumulative)	IPD = 7.102t-4.572	0.970	144.57

As shown in the above table, the rate of increment of cumulative rate of TB cases per 1000 pop, Malaria cases per1000 pop, OPD per 1000 pop and IPD per 1000 pop were 1.68, 7.586, 295 and 7.102 respectively. Here malaria death had higher cumulative rate.

## Discussion

### Mortality and morbidity indicators

There was generally a decreasing trend of all mortality indicators during the study period. As indicated in the results section, all mortality indicators except the maternal mortality rate haven't shown a statistically significant decrease between 1987 and 1998 E.C. This finding is a result of confidence intervals comparison that depends on the sample size considered. However, a decrease of 33 infant deaths per 1000 live births, a reduction of 42 under-five deaths per 1000 children under-five years of age, and a decrement of 5 deaths per 1000 population are of great practical significance for a health system in a developing country. These were in line with the Health policy directions of the Ethiopian government [5-7]

There was high decrement of AIDS related mortality since 1997 E.C as shown in the AIDS mortality graph. This time was also associated with an increment in the number of people living with HIV/AIDS. These changes are most probably due to the introduction of free Anti-retroviral treatment (ART) at that period in time which had resulted in the decrement of number of AIDS related deaths and increment of HIV+ people.

The magnitude of deaths due to Tuberculosis and Malaria had shown a dramatically decreasing trend since 1996 E.C. That decrement trend was higher for malaria than for Tuberculosis. National efforts for malaria and Tuberculosis control programs through the support from the Global fund are most likely the possible explanations for such decrement. The ministry of health, as part of the Millennium Development Goals targets, has given the greatest attention to availing two insecticide treated nets per household and improving the case detection and decreasing the default rates of TB treatment [6].

There was an increasing trend in the number of outpatient visits and inpatient admissions during the study period. Although it is expected to have a decreasing trend the number of new and total Tuberculosis cases had also an increasing trend. It is true that there is an expansion of Health facilities and increasing access to health facilities and an improvement in the health management information system (HMIS) during the study period. It would most likely be due to these factors, rather than a true increment in morbidity that the trend has shown an increasing pattern.

The peak in the number of malaria cases per 100,000 population were in 1991 and 1996 E.C. These peaking effects in the number of malaria cases were due to the malaria epidemics in those years. Plasmodium Falciparum remained to be the dominant malaria species among the diagnosed cases contributing up to 65% of the malaria cases till 1998 E.C. However, that scenario was reversed in 1998 E.C when Plasmodium Vivax diagnosed cases contributed 65% of all malaria diagnosed cases. What has caused this reversal of the two malaria species needs further study. On the other hand the prevalence of mixed infections has increased during the last three years of the study period.

### Health service coverage indicators

Among the Health service coverage indicators EPI and CPR had shown the highest increment during the study period as compared to the other health service coverage indicators considered in this study. This could possibly be due to the several well coordinated immunization campaign coordinated among the ministry of health, World Health Organization, UNICEF and other governmental and non-governmental stakeholders.

On the other hand, the lowest increments among the health service coverage indicators were on skilled birth attendant and postnatal care services coverage. This has been the case in several other cross-sectional studies which claimed cultural factors and perceived quality of the services to be the major contributing factors.

The increments in most health service coverage indicators were smooth till 1998 E.C. However, in 1999 and 2000 E.C there were sharp increments especially in CPR and EPI coverage. This might be due to the deployment of more Health Extension workers to rural health posts who are primarily focusing on promotive and preventive health care services.

### Health system resource indicators

The hospital to population ratio in Ethiopia which is 1:518.948 is higher when compared to the previous values but still lower than that of most African countries. The current Health centre to population ratio which is 1:91,726 is much lower than the expected which is 1:25,000. Based on this expected health centre to population ratio additional 2200 health centres are needed. But the current Health post to population ratio (1:5,198) is very close to the standard (1:5,000).

The Ethiopian Health system has currently a physician to population ratio of 1:22,198 in 2000 E.C. The WHO standard is 1:10,000. Hence the country needs to double the current number of physicians to meet the standard. In contrary to the physician to population ration, the current nurse to population ratio (1:4,519) now exceeds the WHO standard (1:5,000). The trend in nurse to population ratio has showed a decreasing tip in 1999 and 2000 E.C, which may be due to their enrolment in to the accelerated health officers training program. While the expected physician to nurse ratio is 1:2, the current Ethiopian physician to nurse ratio is 1:5.

Had it not been for the non-uniform distribution of physicians in different areas of the country, each hospital would have about 19 physicians, if the currently available physicians were to be equally distributed to all hospitals. Similarly, if all the Health officers are to be equally distributed to all the health centres, each health centre will have at least one health officer. With regard to the health centre to hospital referral, each hospital will have about 6 referring health centres.

During the study period the average increment in total health budget and total Health Expenditure per capita per year were 1.33ETB and 1.1ETB respectively. During the same period, the average increment in GDP per capita per year was 202ETB. Hence one can see that these rates of increment are far apart.

### Determinants of maternal mortality

Total fertility rate, physician per 100,000 population, skilled birth attendance and post-natal care service coverage were found to be determinants of Maternal Mortality Ratio. Among those determinants total fertility rate and physician per 100,000 population have positive association with Maternal Mortality Ratio. However skilled birth attendant and post-natal care coverage associated negatively with Maternal Mortality Ratio.

The decrement of Maternal Mortality Ratio has strong association with Total fertility with β coefficient of 723.6. The decrement in total fertility rate in turn might be due to the increment in contraceptive prevalence rate which is the one which has significant increment among all health service coverage indicators in the study and female literacy rate.

When physician per 100,000 population is correlated with Maternal Mortality Ratio, there was a negative association. However, when other variables were controlled, the negative association is reversed to positive association. This is contrary to the expectation that an increment in physician per 100,000 population is related with a decrement in Maternal Mortality Ratio. What so ever the case, this might be due to the distribution of physicians more in urban areas which might result a decrement of Maternal Mortality Ratio in urban areas only. This might need disaggregated analysis across urban and rural areas.

Skilled birth attendance rate and postnatal care coverage had negative association with Maternal Mortality Ratio. But these are the health service coverage indicators with lowest increment during the study period. To reduce Maternal Mortality Ratio to a greater extent, skilled birth attendance rate and postnatal care coverage should get increased.

### Prediction of indicators for 2015

The MDG targets related to mortality for 2015 include reduction of under-five mortality by two-third and reducing three quarters of Maternal Mortality Ratio. The MDG targets related to morbidity for 2015 are to have halted and begun to reverse the spread of HIV/AIDS; to have halted and begun to reverse the spread of malaria, and to halve the prevalence of tuberculosis. The MDG target related to risk factors for 2015 is to halve the number of people without sustainable access to safe drinking water and basic sanitation (Table 11). These targets has taken in to account the baseline population in 1990 [8].

**Table 11:** Target and predicted values of MDG indicators by 2015 for Ethiopia

S#	MDG indicators	1990	Target by 2015	Projection for 2015
1	Under-five mortality rate	204	68	92

2	Maternal mortality ratio	1400	350	531

3	People with safe drinking water	25	62.5	41

4	People with basic sanitation	8	54	100 by 2011

Although all MDG indicators are targeted for 2015 as an end date to measure success clearly earmarked target values for 2015 were obtained only for the four major indicators. These were the two mortality indicators (Maternal Mortality Ratio, under-five mortality rate) and two risk factor indicators (proportion of people with safe drinking water and proportion of people with basic sanitation).

As indicated in Table 11, the under-five mortality rate in 1990 was 204. The MDG was meant to decrease this value by two-third. This is equivalent to reducing it to 68 (52.4, 83.6). Using the prediction equation obtained in this study, the predicted value for 2015 was 92 (74,110). As the confidence intervals of the target and predicted values are overlapping, it can be assumed that the MDG target for under-five mortality can be achived provided that the current rates of reducing under-five mortality are maintained and/or improved. The decrease of Total Fertility by one might have contributed to the findings [9,10].

On the other hand, achieving the MDG target for reducing maternal mortality by 2015 needs accelarating the current rates of decreament. The MDG intended to reduce the maternal mortality, by then 1400, by three quarters. The expected rate for Ethiopia by 2015 will thus be 350 (313.4, 386.6). The predicted value for 2015 using the equation obtained in this study was 531 (486, 576). As it is evident, these confidence intervals do not have overlapping areas. This indicates that it would be difficult to reach to the MDG target for maternal mortality unless the current rate of decreament is enhanced. However, practical experiences show that the Health Extension program of Ethiopia would facilitate such success.

Access to safe water supply seems on good progress. In 1990 access to safe water supply in Ethiopia was only 25%. Correspondigly, 75% of the population didn't have any access to safe water supply. The MDG target for 2015 was to halve the number of people who didn't have access to safe water supply. It was to increase the access to safe water supply to 62.5% (53%, 72%). The predicted value using the model obtained in this research was 41% (31.4%, 50.6%). A slightest acceleration in the rate of increament could result in the achievement of the MDG target.

More encouraging results were obtained on access to basic sanitation. Access to basic sanitation in 1990 was only 8% showing that about 92% of the population didn't have any access to basic sanitation. The MDG target was to halve this figure i.e. to reduce this 92% to 46%. This was equivalent to increasing access to basic sanitation to 54% by 2015. The good news is that access to basic sanitation will reach 100% (universal access) by 2011 based on the prediction in this study assuming that the current rate of growth is at least maintained.

Using the confidence intervals for projected values and target values, it is clear that the confidence intervals of under-five mortality rate and access to safe drinking water for target and projected values overlap indicating possibility of reaching the targets. However, the confidence interval of Maternal Mortality Ratio for targets and projected values are not overlapping indicating that achieving the target for Maternal Mortality Ratio is less likely provided that the current trend continues. This analysis is based on the measurement methods of the MDG [11].

These findings have several policy implications. Firstly, the goverment should at least maintain the rate at which under-five mortality and access to safe water are improving in order to reach the MDG targets for this indicators. Secondly, the goverment of Ethiopia is less likely to achieve the MDG target for Maternal Mortality Ratio if the rate of decreament is not changed. Hence, there is a need to innovate some mechanisms to abruptly reduce the Maternal Mortality Ratio. This was actually similar to the trends of some of the developing countries [12].

## Conclusions

Based on the findings of the study, the following conclusion are made:

1. Among the mortality indicators considered in this study, only Maternal Mortality Ratio has shown statistically significant change during the study period. Though statistical significance differs from practical significance, it indicates the need to decrease the mortality indicators to a greater extent.

2. Total Fertility Rate, physician per 100,000 population, skilled birth attendance and post-natal care service coverages were found to be determinants of the decrease in Maternal Mortality Ratio. While TFR and Physician per 100,000 population are positively associated, skilled birth attendance and PNC are negatively associated with MMR.

3. Plasmodium Falciparum was the leading malaria species till 1998. But there was a reversal of malaria parasite prevalence in 1999 E.C from P. Falciparum to P. Vivax. Mixed infection of both P. Falciparum and P. Vivax also shows an increasing trend.

4. Health Service coverage indicators showed a remarkable improvement during the study period. Despite they are the key determinants of MMR, skilled birth attendant and post-natal care coverage have the least increment with a rate of 1.24 and 1.42 per year respectively among the Health Service coverage indicators.

5. The MDG target for proportion of people having access to basic sanitation can be achieved 100% by 2011. However, Maternal Mortality Ratio, Under-five mortality rate, and proportion of people with safe and clean water MDG targets might not be reached if the current trend is assumed to continue.

## Lists of abbreviations

AIDS: Acquired Immune deficiency syndrome; E.C: Ethiopian Calendar; ETB: Ethiopian Birr; GDP: Gross Domestic Product; HIV: Human Immune virus; MDG: Millennium Development Goals; OPD: Outpatient Department; PoP: Population; TB: Tuberculosis; TFR: Total Fertility Rate; USAID: United States Agency for International Development; WHO: World Health Organization

## Competing interests

The authors declare that there are no competing interests

## Authors' contributions

Both Authors are involved in all the steps from the inception of the research idea to the preparation of the manuscript. MAW has involved in writing the protocol, research tools, data collection, and analysis and writes up of final reports. TN has involved in reviewing the protocol, data collection tools and reports. Both of the authors involved in manuscript preparation.

## Authors' information

MAW is Health promotion and Disease prevention officer at the Federal Ministry of Health of Ethiopia. She has an MPH degree and years of experience in the health system. TN is a Quality assurance specialist for HIV/AIDS programs in the United States Agency for International Development (USAID/Ethiopia) with experiences in Health system researches and evaluations.

## Appendix 1

### Data sources

#### Ministry of Health

The Ministry of Health yearly publications of Health and Health related indicators from 1987 to 2000 E.C were the major guiding documents in this study. Besides, the AIDS reports from 1990 E.C to 1998 E.C were also considered. The TB database from the Ministry of Health was also used.

#### Central Statistical Authority

Two census reports (1994 and 2007) and two Demographic and Health survey reports (2000 and 2005) were used. The Health and Nutrition surveys and the welfare survey reports of the authority were also consulted for this study. Statistical abstracts were also used.

#### Ministry of Education

The Educational Management information system (EMIS) reports (Educational statistics were also used to obtain the literacy indicators that could affect the Morbidity and mortality indicators of the country. The indicator values from these sources were taken as the basic sources of education related indicators.

#### Ministry of Finance and Economic Development

The Ministry of Finance and Economic Development (MOFED) was the source of Economic indicators like the Gross Domestic Product (GDP) and Gross National Product (GNP) per capita. The reports of this ministry on MDG status were also used.

#### Ministry of Water Resources Development

The coverage of safe water supply and sanitation for the more recent years was obtained from the Ministry of Water Resources Development (MoWRD). However, the older data were on water and sanitation was from the Ministry of Health.

#### Ethiopian Health and Research Institute (EHNRI)

The documents from this institute were on nutritional and laboratory based indicators.

#### World Health Organization

World Health Report from 1996 to 2008 was explored for the indicators of Ethiopia. Besides, the World Health organization statistical information system is browsed for specific Health and Health related indicators.

#### World Bank

The World Bank World development reports from 1996 to 2008 were scrutinized for several development related indicators for Ethiopia. These indicators were also quoted by the Health and Health related indicators of Ethiopia.

#### UNAIDS

The AIDS Epidemic updates of UNAIDS were also consulted for national figures on HIV and AIDS related issues. The data from these sources were also seen in relation with the data from the national HIV/AIDS publications by the Ministry of Health.

#### UNICEF

The publication of UNICEF about the country profile of Ethiopia was taken in to consideration for some important indicators in Ethiopia. However, the indicators are only the most recent for use by the organization.

#### UNDP

The Millennium Development Goal reports for Ethiopia from the worldwide updates were also consulted to cross check some of the important indicators in Ethiopia. However, these are only for a few numbers of years as published by the UN team.

#### USAID

The Country update of USAID for Ethiopia in 2007 bears the major recent Health and Health related indicators of Ethiopia. These indicators were taken together with other recent indicators of other international and national sources.

### Appendix 2

#### Data quality assurance

All the data collection process, entry and cleaning were done with the direct involvement of the principal investigator. Backup copies were saved for each step of the work. All the indicator values were double checked against their values in the indicator document. However, the following problems were faced and solutions were attempted.

#### Inconsistent values

There were some inconsistencies among the indicators values between national and international sources. There were also some discrepant values among national sources too. Even different values of the same indicators on the same source were observed at different reports of different times, years. When there were differences as indicated above, the most proximate data source or reporting period was assumed to be the more reliable data and included in this study. In cases where level of proximity of the data sources or the reporting period to the data is similar, the mandate and expertise of the reporting body on the specific indicators was used.

#### Incomplete values

As indicated in the documents themselves, some indicators values were reported for some part of the country only i.e. the indicator values do not include some regions. For example in the Health and Health related indicators of 1992 E.C for proportion of fully immunized under-one children doesn't include the report from Addis Ababa city administration Health bureau. In situations where better sources of indicator values can be obtained, such data sources were used. As the ministry and other national decision making bodies were and are using this type of data, this study considered the inclusion of the data in the analysis. However, the incompleteness of the data is considered in the interpretation of the results.

#### Missing values

Despite the rigorous exploration of the various data sources, some indicators values can't be obtained. However, the absence of the evidence may not be the evidence of absence of the data. Different hierarchies of data exploration were used before declaring the data as missing. When the magnitude of missed values is minimal the values were not considered in the analysis. For some of the indicator values, replacement was done by back and forth forecasting based on the equation derived from the existing values of the indicator.

#### Recent introduction

Some of the Health and Health related indicators were introduced very recently and complete values can't be obtained. Even some indicators were excluded from the report in the recent years. An example in the Health extension workers are reported as of 1997 E.C. The indicators were analyzed using the existing values only. These indicators are included because the stakeholders included the new indicators because either it is found very relevant and/or it can be measured within the available resources. Just for the purpose of the analysis some valued were filled using back projections.

#### Changes in measurement

Some indicator values showed measurement changes across the study years. For instance, the number of HIV/AIDS surveillance sites was increased and the projection method was changed to spectrum model. There was also shifting of responsible bodies that might result in changes in measurement of the indicators such as water supply shift from MOH to MoWRD. In such cases, the changes in measurement were to improve the quality of data. That is to increase representativeness, reliability, validity and coverage of the indicators values. As these changes can have effects on the results of this study, the changes were considered in the interpretation of the results.

## References

[B1] Canadian Institute for Health Information (October 2001), Developing environmental public health indicators in Canada, Paper submitted by Environment Canada - Health Canada

[B2] Federal Ministry of Health, Ethiopia (2006/7), Health and Health related indicators, Addis Ababa, Ethiopia

[B3] Health policy of the transitional government of Ethiopia (Sept. 1993)

[B4] Federal Ministry of Health, Ethiopia (2007), The HSDP Harmonization Manual (HHM) 1

[B5] Federal Ministry of Health, Ethiopia (2005), Health Sector development plan (HSDP III) 2005/6-2009/10, Addis Ababa, Ethiopia

[B6] Central Statistical Agency [Ethiopia] and ORC Macro Ethiopia Demographic and Health Survey 2005 2006 Addis Ababa, Ethiopia and Calverton, Maryland, USA: Central Statistical Agency and ORC Macro

[B7] Central Statistical Authority [Ethiopia] and ORC Macro Ethiopia Demographic and Health Survey 2000 2001 Addis Ababa, Ethiopia and Calverton, Maryland, USA: Central Statistical Authority and ORC Macro

[B8] Central Statistical Authority (CSA) [Ethiopia] The 1984 Population and Housing Census of Ethiopia Analytical Report at National Level 1991 Addis Ababa, Ethiopia: Central Statistical Authority

[B9] Central Statistical Authority (CSA) [Ethiopia] The 1990 Family and Fertility Survey-Preliminary Report 1991 Addis Ababa, Ethiopia: Central Statistical Authority

[B10] Central Statistical Authority (CSA) [Ethiopia] The 1994 Population and Housing Census of Ethiopia. Results at Country Level Statistical Report 1998 1 Addis Ababa, Ethiopia: Central Statistical Authority

[B11] World Health Organization (2003), the Millennium Development Goals, The health indicators: scope, definitions and measurement methods, Geneva

[B12] World Health Organization (2008), World Health Statistics, France

